# Stochastic process and tutorial of the African buffalo optimization

**DOI:** 10.1038/s41598-022-22242-9

**Published:** 2022-10-15

**Authors:** Julius Beneoluchi Odili, A. Noraziah, Basem Alkazemi, M. Zarina

**Affiliations:** 1 Faculty of Science and Science Education, Anchor University Lagos, Lagos, Nigeria; 2 Faculty of Computing, Universiti Malaysia Pahang, Pekan, Kuantan Malaysia; 3 Centre for Software Development and Integrated Computing, University Malaysia Pahang, 26600 Pekan,, Pahang Malaysia; 4 Department of Computer Science, Umm al-Qura University, Makkah al Mukarramah, Saudi Arabia; 5 Faculty of Informatics and Computing, Universiti Sultan Zainal Abidin, Kuala Terengganu, Malaysia

**Keywords:** Computational biology and bioinformatics, Mathematics and computing

## Abstract

This paper presents the data description of the African buffalo optimization algorithm (ABO). ABO is a recently-designed optimization algorithm that is inspired by the migrant behaviour of African buffalos in the vast African landscape. Organizing their large herds that could be over a thousand buffalos using just two principal sounds, the /maaa/ and the /waaa/ calls present a good foundation for the development of an optimization algorithm. Since elaborate descriptions of the manual workings of optimization algorithms are rare in literature, this paper aims at solving this problem, hence it is our main contribution. It is our belief that elaborate manual description of the workings of optimization algorithms make it user-friendly and encourage reproducibility of the experimental procedures performed using this algorithm. Again, our ability to describe the algorithm’s basic flow, stochastic and data generation processes in a language so simple that any non-expert can appreciate and use as well as the practical implementation of the popular benchmark Rosenbrock and Shekel Foxhole functions with the novel algorithm will assist the research community in benefiting maximally from the contributions of this novel algorithm. Finally, benchmarking the good experimental output of the ABO with those of the popular, highly effective and efficient Cuckoo Search and Flower Pollination Algorithm underscores the ABO as a worthy contribution to the existing body of population-based optimization algorithms

## Introduction

The obvious contributions of optimization to ensuring efficiency and effectiveness of industrial and engineering processes have led to the popularity of optimization as an indispensable subfield in artificial intelligence, computer science and engineering fields of study. Optimization has been described as the economics of science and engineering^1^. This definition is apt because optimization is concerned with the minimization of inputs in order to obtain maximum possible yield. It finds relevance in basically all aspects of science and engineering. In industrial production, for instance, manufacturing and process engineers are concerned with the optimization of available resources (raw materials, industrial machines, human resources and time) in order to yield the greatest number of finished products of acceptable quality^2^. Optimization continues even to the distribution of such finished products. The transportation of the finished products to the distributors, customers and end-users should be done in a way that will maximally benefit the organization in terms of time and cost. Even at the level of end-users, optimization is required by the consuming organizations cum individuals in their use of the finished products to meet their organizational/individual needs. From the foregoing discussion, the need for optimization cannot be over-emphasized^3^.

This overbearing influence of optimization has led to the development of several optimization algorithms in an attempt to improve the optimization procedures. Some of the popular optimization algorithms in literature include Genetic Algorithm, Particle Swarm Optimization, Sine-Cosine optimization algorithm^4^, Simulated Annealing, Hill Climbing, Tabu Search, Hybrid Whale Nelder Mead algorithm^5^, Great Deluge Algorithm etc^6^. These algorithms have been applied to solve several optimization problems ranging from vehicle routing, network routing, constrained truss optimization problems^7^, job scheduling, collision-avoidance^8^, mobile ad-hoc networks, tuning PID parameters of Automatic Voltage Regulators^9^, sports and examination timetabling^10^, test suite optimization^11^, energy enhancement^12^, global optimization problems^5^, automobile connecting rod components etc^13^ with good results.

The constant need for further improvements in the existing technologies has led to the development of some recent development algorithms. These newly-developed optimization algorithms are sometimes called twenty first century algorithms. Notable among these twenty first century algorithms are the Firefly, Ebola Optimization Search Algorithm^14^, Reptile Search Algorithm^15^, Bat Algorithm^16^, Dwarf Mongoose Optimization Algorithm^17^, Animal Migration Optimization^18^, Grey Wolf Optimization^19^, League Championship^20^, Sine–Cosine, Water Cycle, Grasshopper, Harris Hawks Optimization, Dragon Fly Optimization Algorithm^21^, Whale Optimization^22^, Gaining-sharing knowledge based algorithm^23^ and the African Buffalo Optimization^24^ algorithms.

Since its introduction, the African Buffalo Optimization has enjoyed warm acceptance among researchers and wide application to different scientific and engineering processes^25–28^. So far, some of the application areas of the African Buffalo Optimization include Strategic management, numerical function evaluation^29^, travelling salesman’s problem, PID parameter tuning of automatic voltage regulators^30^, collision avoidance in electric fish^31^ strategic integration of battery energy storage in distributed networks^28^ etc. This warm reception necessitated the need for a detailed explanation of the algorithm’s operational processes so that other researchers may better understand its internal mechanisms and further explore the strengths of the algorithm. This is the motivation for this study. This study is significant because it is rare to find a similar study highlighting the literal working of newly-designed algorithms from a human perspective. Instead what is available, in literature, is a brief description from a machine perspective^32^. It is hoped that this study will simplify software design and development using the African Buffalo Optimization algorithm in solving different engineering, scientific and industrial problems. Moreover, it is hoped that it will stimulate interest, among researchers, in highlighting the literal working of algorithms from a human perspective. The knowledge of the literal workings of the African Buffalo Optimization algorithm will enhance the understanding, implementation and use of the algorithm.

The rest of this paper is organized as follows: section two examines the materials and methods that basically describes the algorithms’ developmental processes as it relates specifically to the ABO; section three discusses the ABO algorithm; section four presents ABO solutions to global optimization problems as well technically exploring ABO’s search procedure in a two-dimensional search space; section five discusses the implementation of the ABO and the CS to solve the benchmark Rosenbrock function specifically highlighting the effects of the number of search population cum iterations in the search process and section six examines the ABO and FPA in solving the benchmark Dejong 5 (Shekel) function. Section seven draws conclusion on the study.

## Materials and methods

The African buffalo optimization (ABO) was inspired by the migrant behaviour of the African buffalos, especially the organizational prowess of the buffalo herd in their movements from one part of Africa to the other in search of grazing pastures^33^. The development of the ABO began with a careful study of the movement and organization of the African buffalos in existing literature as well as from television documentary programs on National Geographic Wild Channel^34,35^.

The design of the ABO is an effort to design a fast, robust, effective, efficient, yet simple-to-implement and user-friendly algorithm imbued with sufficient capacity to exploit and explore the solution space through thorough simulation of the democratic cum communicative capabilities of cape buffalos, (otherwise called African buffalos) in their quest for solutions^36^.

ABO simulates the cooperative nature, communicative acumen coupled with the communal decision-making procedures of the African buffalos that places much premium on the harnessing of the collective intelligence of the entire herd. The buffalos use mainly two vocalizations to organize themselves in their search for solutions: the attraction sound */maaa/* for exploitation and repulsion /*waaa/* sound for exploration. The herd movement, provision and protection of the entire buffalo community hinges on the effective utilization utilisation of both calls.

### Stages in the development of the ABO

As earlier observed, the design of a swarm intelligence algorithm follows a six-step procedure. These six steps were diligently followed in the design of the African Buffalo Optimization^37,38^, namely:

i. Careful observation of the behavior of a group of organisms/creatures working harmoniously to realize the group’s objectives that seem rather impossible for an individual member of the group. The African buffalos were keenly studied based on observation from the National Geographic Wild television channel
ii. A model was developed that fully describes the behavior of a herd community, in this case of the African buffalos
iii. The development of a mathematical model based on the model of behavior developed in (ii)
iv. A pseudocode was designed to simulate the behavior of African buffalos
v. A programming code was developed to implement the pseudocode
vi. The programming code was subjected to various mathematical cum experimental evaluations with the aim of fine-tuning the algorithm’s parameters to achieve the set objectives^39^.

**Figure 1:**
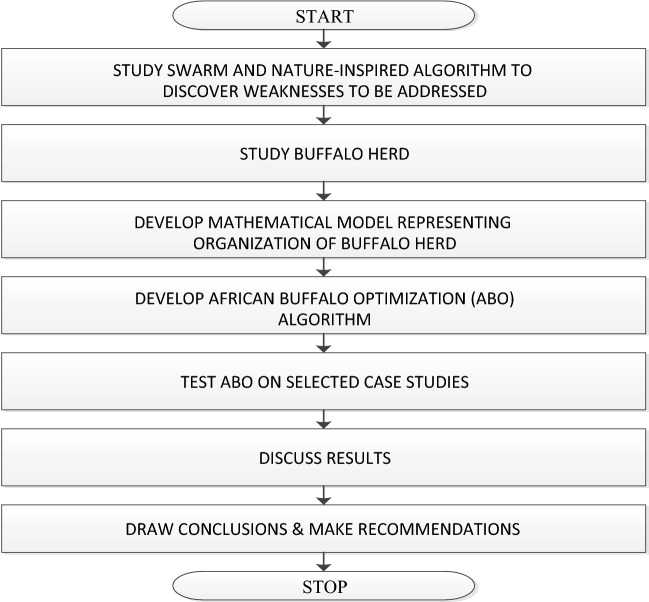
ABO design methodology flowchart.

The methodology for design and applications of the ABO algorithm is presented in Fig. 1.

## The ABO algorithm

**Figure 2:**
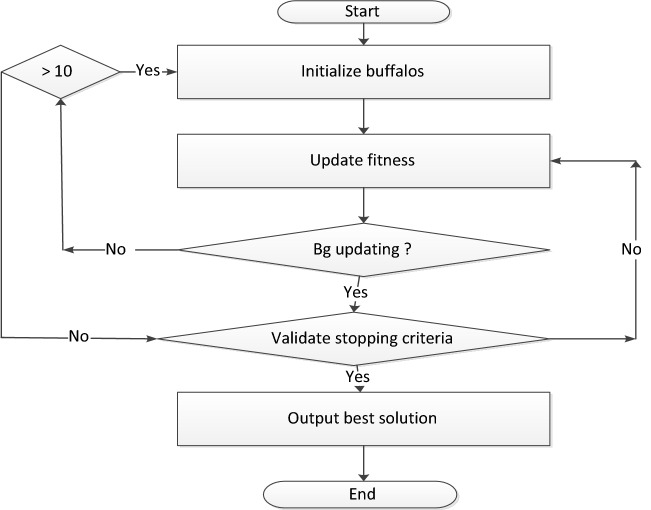
ABO algorithm.


The ABO algorithm^40^ and flowchart is presented in Fig. 2:

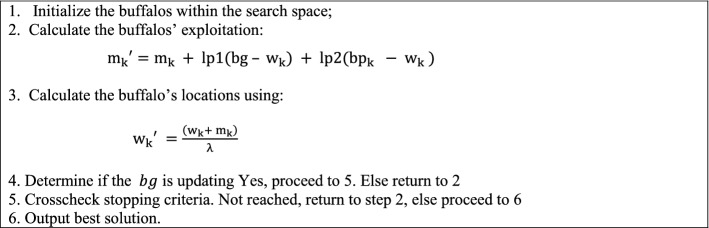


Please note that in Fig. 2, ${\mathrm{w}}_{\mathrm{k}}$ represents the /*waaa*/call. This call mobilizes the herd to move on (explore) with particular reference to buffalo k. The ${\mathrm{m}}_{\mathrm{k}}$ represents the /*maaa*/ call to exploit. Similarly, ${\mathrm{w}}_{\mathrm{k}}^{\mathrm{^{\prime}}} ,$ the call for more exploration; ${\mathrm{m}}_{\mathrm{k}}^{\prime}$, a represents need for more exploitation; $\mathrm{lp}1$ and $\mathrm{lp}2$, the learning parameters; and $\lambda$ a random number which takes any value between 0 and 1 depending on the problem being solved: the higher the value, the more the exploitation and less of exploration and vice-versa.

### ABO mathematical description

The ABO starts by randomly initializing the buffalo population within the search space. Next the buffalo’s exploitation capacities are evaluated using Eq. (1). The outcome of this evaluation is crucial in determining the next move of the buffalos in their search for fruitful grazing locations. The result of democratic Eq. (1) is fed into the exploration Eq. (2) [see Eq. (2) below] to determine whether the buffalos will remain in the same location or migrate to another location. If the $bg$ (the buffalo with the best position in relation to the global optimum) is updating, the algorithm verifies if the stopping criteria has been reached. If yes, it terminates the run and outputs the location of the best buffalo as the output. If the stopping criterion has not been reached, the algorithm returns to step 2 to reassess the buffalos’ exploitation values.

The controlling equation that propels the entire buffalo herd to relocate to other locations, probably more rewarding than the present location is:

1 $$ {\text{m}}^{\prime } _{{\text{k}}}  = {\text{m}}_{{\text{k}}}  + {\text{lp}}1\left( {bg - {\text{w}}_{{\text{k}}} } \right) + {\text{lp}}2\left( {bp_{k}  - {\text{w}}_{{\text{k}}} } \right) $$

Equation 1 which is the exploration equation has three parts: the memory part (${\mathrm{m}}_{\mathrm{k}}{^{\prime}}$) that reminds the buffalos that they have relocated to a new location from the previous location $({\mathrm{m}}_{\mathrm{k}}$); the second part which signals the cooperative behaviour of the buffalos$, (\mathrm{lp}1\left(bg - {\mathrm{w}}_{\mathrm{k}}\right))$ and the third part ($\mathrm{lp}2\left({bp}_{k}-{\mathrm{w}}_{\mathrm{k}}\right)$) represents the buffalos capacity for excellent communication among the entire herd. Note that this decision is influenced by the learning parameter $\mathrm{lp}1$.

The last part of Eq. (1): ($\mathrm{lp}2\left({bp}_{k}- {\mathrm{w}}_{\mathrm{k}}\right),$ underscores the exceptional intelligence of these animals. They can tell their previous best productive location in comparison with their present location. This knowledge enables the buffalos to retrace their steps to the best previous rewarding location whenever they stray away into a starving location. Also, note that the buffalo’s exceptional intelligence with regards to the previous best rewarding location vis-à-vis their present location is influenced by the learning parameter $\mathrm{lp}2.$ As can be observed, the entire Eq. (1) highlights the buffalos’ ability to harness the collective intelligence, excellent memory capacity cum regular communication of the herd in making informed decisions in their search for solutions^41^.

Again, it can be observed that the algorithm subtracts the dimensional element ${\mathrm{w}}_{\mathrm{k}}$ from the maximum vector and then multiplies this by the learning parameters ($lp1$_,_ $lp2$) usually between 0 and 1. The right values of the learning parameters ($lp1$_,_ $lp2$) are obtainable through parameter tuning process. Please note also that a higher value of $lp1$ biases the search towards global search while the higher the value of $lp2$, the local search. The sum of these products is then added to the exploitation memory part of the equation (${\mathrm{m}}_{\mathrm{k}}$) for the given dimension ($x or y$) to determine the actual fitness of the buffalos. Equation 2 basically, propels the buffalos to a new location following the outcome of Eq. (1).

2 $${\mathrm{w}}_{\mathrm{k}}{^{\prime}} =\frac{\left({\mathrm{w}}_{\mathrm{k}}+ {\mathrm{m}}_{\mathrm{k}}\right)}{\lambda }$$

**Figure 3:**
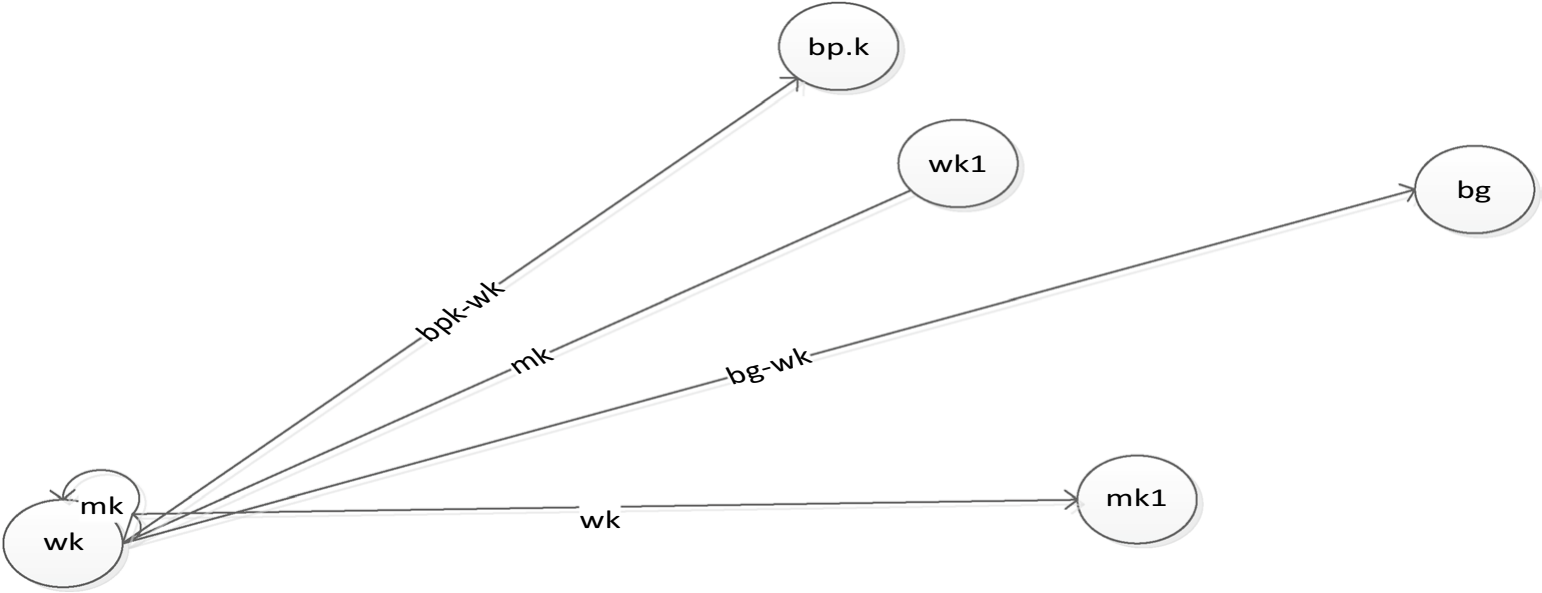
ABO visualization.

From Eq. (2), it can be seen that the movement of the buffalos is a function of the /waaa/ calls (${\mathrm{w}}_{\mathrm{k}}$) and the /maaa/ $\left({\mathrm{m}}_{\mathrm{k}}\right)$ calls of the buffalos being moderated by exploration driver $\lambda$ which takes a value between 0 and 1. The higher the value of the $\lambda$, the less exploration and vice-versa. The ABO can be visualized thus:

In Fig. 3, the movement of buffalo $k$, therefore, from ${\mathrm{w}}_{\mathrm{k}}$ (the present exploration location), to other locations has to be influenced by other factors such as the ${\mathrm{m}}_{\mathrm{k}}$, the exploitation location and appropriate adjustment of its position in relation to the herd’s best $(\mathrm{bg } - {\mathrm{w}}_{\mathrm{k}})$ as well as its personal best (${\mathrm{bp}}_{\mathrm{k}} - {\mathrm{w}}_{\mathrm{k}})$ with the covert bias of the learning parameters.

## ABO for global optimization problems

In modern scientific investigations, scientists encounter problems that are multimodal with diverse objective functions and having different channels, hyperplanes, valleys, peaks etc. Ability to provide solutions to such global optimization problems distinguishes an effective and efficient optimization algorithm from the rest^42^. In our attempt to unravel the search potentials of the ABO, it is necessary to simulate ABO search procedure in a two-dimensional search space but first, let us examine the ABO solution steps.

### ABO steps for solving global optimization problems

1. Initialize the buffalos randomly within the search space
2. State the controlling ABO learning parameters: $lp1\; and\; lp2$
3. Using Eq. (1), verify the herd exploitation state noting each buffalo’s $bp$ and the $bg$ for the entire herd
4. Using Eq. (2), determine the location of the buffalos
5. Check if the $bg$ is updating. Yes, go to Step 6, else return to Step 2
6. Verify stopping criteria. Reached, go to Step 7, else go to Step 3
7. Output the best result.

### ABO on a two-dimensional space

**Figure 4:**
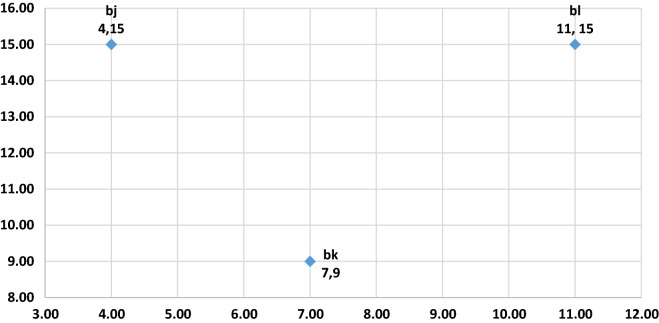
Starting locations.

At this juncture, let us attempt the demonstration of ABO on a two-dimensional search space. For this exercise, we initialize buffalo $k$ to location 7, 9; buffalo $l$ to 11, 15 and buffalo j, 4, 15 (see Fig. 4). Again, we assume the global optimum point is 41.5, 75. In the first iteration, we place $lp1$ as 0.6, $lp2$ as 0.5, $\lambda$ is a random number [0, 1]. It is important to observe that the lower the value of $\lambda$, the more the exploration and vice-versa. For the sake of convenience, let $\lambda$ assume values between 0.5 and 0.9 (see Fig. 1).

*Iteration 1 (for* $${b}_{k}$$ *):*

$bp$ =each buffalo’s starting points,

$bg$ =11,15(bj), picked randomly

$${\mathrm{m}}_{\mathrm{k}}{^{\prime}}={\mathrm{m}}_{\mathrm{k}}+\mathrm{ lp}1(bg - {\mathrm{w}}_{\mathrm{k}})+\mathrm{lp}2(bp.\mathrm{k}-{\mathrm{w}}_{\mathrm{k}})$$

$$ \begin{aligned} {\text{m}}^{\prime }_{{\text{k}}}  (x) & { = }{7} + 0.6\left( {{11}{-}{7}} \right) + 0.5*\left( {{7} - {7}} \right) \\ & = {7} + 0.6\left( {4} \right) + 0.5\left( 0 \right) \\ & = {7} + {2}.{4} + 0 \\ &{\text{m}}^{\prime }_{{\text{k}}}(x) = 9.4 \\ \end{aligned} $$

$$ {\text{m}}^{\prime }_{{\text{k}}} = {\text{m}}_{{\text{k}}} + {\text{lp}}1\left( {bg{-}{\text{w}}_{{\text{k}}} } \right) + {\text{lp}}2\left( {bp.{\text{k}} - {\text{w}}_{{\text{k}}} } \right) $$

$$ \begin{aligned} {\text{m}}^{\prime }_{{\text{k}}} \left( {\text{y}} \right) & = {9} + 0.6\left( {{15} - {9}} \right) + 0.5\left( {{9} - {9}} \right) \\ & = {9} + 0.6\left( {6} \right) + 0.{5 }\left( 0 \right) \\ & = {9} + {3}.{6} + 0 \\ &{\text{m}}^{\prime }_{{\text{k}}} (y) = 12.6\\ \end{aligned} $$

$$ {\text{hence}},\;{\text{m}}^{\prime }_{{\text{k}}}  = \left( {{9}.{4},{12}.{6}} \right). $$

At this point, the present exploitation values of buffalo k, represented by ${\text{m}}^{\prime }_{{\text{k}}}$ is (9.4, 12.6). Next, let us apply these new exploitation fitness dimensions to our buffalo location using exploration decision Eq. (2)

$$ {\text{w}}^{\prime }_{{\text{k}}} = \frac{{\left( {{\text{w}}_{{\text{k}}} + {\text{m}}_{{\text{k}}} } \right)}}{\lambda } $$

$$ \begin{aligned} {\text{w}}^{\prime }_{{\text{k}}} (x) & = \frac{{\left( {7 + { }9.4} \right)}}{0.5} \\ & = 32.8 \\ \end{aligned} $$

$$ \begin{aligned} {\text{w}}^{\prime }_{{\text{k}}}(y) & = \frac{{\left( {9 + { }12.6} \right)}}{0.5} \\ & = 43.2 \\ \end{aligned} $$

$$ {\text{hence}},\;{\text{w}}^{\prime }_{{\text{k}}}  = \left( {{32}.{8},{43}.{2}} \right) $$

**Figure 5:**
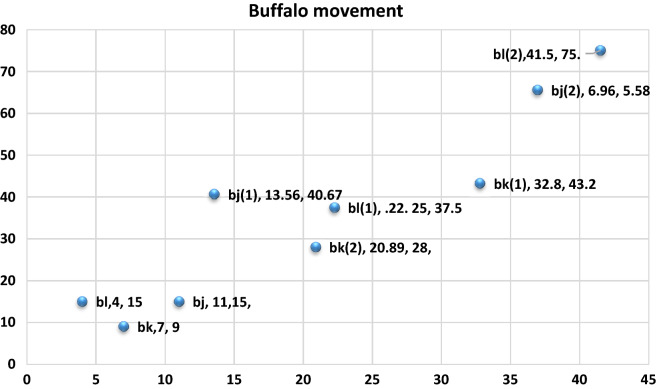
1st Iteration.

Also, please note that the present location of buffalo $k$, represented by ${\text{w}}^{\prime }_{{\text{k}}}$ is (32.8, 43.2). Plot these new values into our graph (see Fig. 5):

Let us now see the performance of the ABO for buffalo $$ l$$ at the first iteration.

*Iteration 1 (for* $$b_{j}$$*):*

$bp$ = each buffalo’s starting points, $bg$ = 11,15($b_{j}$), picked randomly

$$ {\text{m}}^{\prime }_{{\text{j}}}  = {\text{m}}_{{\text{j}}} + {\text{lp}}1\left( {bg{-}{\text{w}}_{{\text{j}}} } \right) + {\text{lp}}2\left( {bp.{\text{j}} - {\text{w}}_{{\text{j}}} } \right) $$

$$ \begin{aligned} {\text{m}}^{\prime }_{{\text{j}}} \left( x \right) & = {4} + 0.6\left( {{11}{-}{4}} \right) + 0.5*\left( {{4} - {4}} \right) \\ & = {4} + 0.6\left( {7} \right) + 0.5\left( 0 \right) \\ & = {4} + {4}.{2} + 0 \\ &{\text{m}}^{\prime }_{{\text{j}}} \left( x \right) = 8.2 \\\end{aligned} $$

$$ {\text{m}}^{\prime }_{{\text{j}}} = {\text{m}}_{{\text{j}}} + {\text{lp}}1\left( {bg{-}{\text{w}}_{{\text{j}}} } \right) + {\text{lp}}2\left( {bp.{\text{j}} - {\text{w}}_{{\text{j}}} } \right) $$

$$ \begin{aligned} {\text{m}}^{\prime }_{{\text{j}}}\left( y \right) & = {15} + 0.6\left( {{15} - {4}} \right) + 0.5\left( {{15} - {15}} \right) \\ & = {15} + 0.6\left( {{11}} \right) + 0.5\left( 0 \right) \\ & = {15} + {6}.{6} + 0 \\ \end{aligned} $$

$$ {\text{m}}^{\prime }_{{\text{j}}} \left( y \right) = 2.16\quad new\;{\text{m}}_{{\text{j}}} = \left( {{8}.{2},{21}.{6}} \right). $$

At this point, the present exploitation values of buffalo $j$, represented by new ${\text{m}}_{{\text{j}}}$ is (8.2, 21.6). Next, let us apply these new exploitation fitness dimensions to our buffalo location using exploration decision Eq. (2)

$$ {\text{w}}^{\prime }_{{\text{j}}} \left( x \right) = \frac{{\left( {{\text{w}}_{{\text{j}}} + {\text{ m}}_{{\text{j}}} } \right)}}{\lambda } $$

$$ \begin{aligned} {\text{w}}^{\prime }_{{\text{j}}} \left( x \right) & = \frac{{\left( {4 + { }8.2} \right)}}{0.9} \\ & = 13.56 \\ \end{aligned} $$

$$ \begin{aligned} {\text{w}}^{\prime }_{{\text{j}}} \left( y \right) & = \frac{{\left( {15{ } + { }21.6} \right)}}{0.9} \\ & = {4}0.{67}\quad {\text{new}}\;{\text{w}}_{{\text{j}}} = \left( {{13}.{56},{ 4}0.{67}} \right) \\ \end{aligned} $$

The new location of buffalo $b_{j}$ is (13.56, 40.67) as shown in Fig. 5.

*Iteration 1 (for* $${b}_{l}$$ *):*

$bp$ = each buffalo’s starting points, $bg$ =11, 15(${b}_{j}$), picked randomly

$$ {\text{m}}^{\prime }_{{\text{l}}}  = {\text{m}}_{{\text{j}}} + {\text{lp}}1\left( {bg - {\text{w}}_{{\text{l}}} } \right) + {\text{lp}}2\left( {bp.{\text{l}} - {\text{w}}_{{\text{l}}} } \right) $$

$$ \begin{aligned} {\text{m}}^{\prime }_{{\text{l}}}  \left( x \right) & = {11} + 0.6\left( {{4} - {11}} \right) + 0.5*\left( {{11} - {11}} \right) \\ & = {11} + 0.6\left( { - {7}} \right) + 0.5\left( 0 \right) \\ & = {11} + - {4}.{2} + 0 \\ & {\text{m}}^{\prime }_{{\text{l}}}  \left( x \right) = 6.8\\\end{aligned} $$

$$ {\text{m}}^{\prime }_{{\text{l}}}  = {\text{m}}_{{\text{j}}} + {\text{lp}}1\left( {bg{-}{\text{w}}_{{\text{l}}} } \right) + {\text{lp}}2\left( {bp.{\text{l}} - {\text{w}}_{{\text{l}}} } \right) $$

$$ \begin{aligned} {\text{m}}^ {\prime }_{{\text{l}}} \left( y \right) & = {15} + 0.6\left( {{15} - {15}} \right) + 0.5\left( {{15} - {15}} \right) \\ & = {15} + 0.6\left( 0 \right) + 0.5\left( 0 \right) \\ & = {15} + 0 + 0 \\&{\text{m}}^{\prime }_{{\text{l}}}  \left( y \right) = 15\quad new\;{\text{m}}_{1} = \left( {6.8,15} \right).\\ \end{aligned} $$

At this point, the present exploitation values of buffalo $l$, represented by new ${\mathrm{m}}_{1}l$ is (6.8, 15). Next, let us apply these new exploitation fitness dimensions to our buffalo location using exploration decision Eq. (2)

$$ {\text{w}}^{\prime }_{{\text{l}}}  \left( x \right){ } = \frac{{\left( {{\text{w}}_{{\text{l}}} + {\text{ m}}_{{\text{l}}} } \right)}}{\lambda } $$

$$ \begin{aligned} {\text{w}}^{\prime }_{{\text{l}}} \left( x \right) & = \frac{{\left( {11 + { }6.8} \right)}}{0.8} \\ & = {22}.{25} \\ \end{aligned} $$

$$ \begin{aligned} {\text{w}}^{\prime }_{{\text{l}}}  \left( y \right) & = \frac{{\left( {15{ } + { }15} \right)}}{0.8} \\ & = {37}.{5}\quad {\text{new}}\;{\text{w}}_{1} = \left( {22.25,37.5} \right) \\ \end{aligned} $$

Now, buffalo $$ b_{l}$$ is in location 22.25, 37.5 (see Fig. 5).

*Iteration 2 (for* $$b_{k}$$*):*

We shall use the dimensions obtained from the first iteration to update our algorithm using Eq. (1). It should be observed that the algorithm updates $$bg$$ value from iteration to iteration. Let us randomly take the $$ b_{k}$$ as the $$bg$$ and the individual buffalo’s newest locations as their $$ b_{p}$$. This is in appreciation of their performances at the first iteration and in line with the ABO’s strategy to avoid stagnation. For iteration2, therefore, the $$bg$$ is 8.9, 15.

$bp$ = each buffalo’s previous points, $bg$ = 32.8,43.2 ($b_{k } (1)$), picked randomly

$$ {\text{m}}^{\prime \prime }_{{\text{k}}}  = {\text{m}}^{\prime }_{{\text{k}}}  + {\text{lp}}1\left( {bg{-}{\text{w}}^{\prime }_{{\text{k}}} } \right) + {\text{lp}}2\left( {bp.{\text{k}} - {\text{w}}^{\prime }_{{\text{k}}} } \right){ } $$

$$ \begin{aligned} {\text{m}}^{\prime \prime }_{{\text{k}}} (x) & = {9}.{4} + 0.6\left( {{32}.{8} - {32}.{8}} \right) + 0.5\left( {{32}.{8} - {32}.{8}} \right) \\ & = {9}.{4} + 0.6\left( 0 \right) + 0.5\left( 0 \right) \\ & = {9}.{4} + 0 + 0 \\ & = 9.4 \\ \end{aligned} $$

$$ \begin{aligned} {\text{m}}^ {\prime \prime }_{{\text{k}}}(y) & = {12}.{6} + 0.6\left( {{43}.{2} - {43}.{2}} \right) + 0.5\left( {{43}.{2} - {43}.{2}} \right) \\ & = {12}.{6} + 0.6\left( 0 \right) + 0.5\left( 0 \right) \\ & = {12}.{6} + 0 + 0 \\ & = {12}.{6} \\&{\text{m}}^{\prime \prime }_{{\text{k}}}  = \left( {{9}.{4},{12}.{6}} \right). \\ \end{aligned} $$

Applying the new exploitation fitness dimensions to our buffalo location using exploitation decision Eq. (2)

$$ {\text{w}}^{\prime \prime }_{{\text{k}}}  \left( x \right) = \frac{{\left( {{\text{w}}_{{\text{k }}} + {\text{ m}}_{{\text{k}}} } \right)}}{\lambda } $$

$$ \begin{aligned} {\text{w}}^{\prime \prime }_{{\text{k}}}  \left( x \right) & = \frac{{\left( {9.4 + { }9.4} \right)}}{0.9} \\ & = {2}0.{89} \\ \end{aligned} $$

$$ \begin{aligned} {\text{w}}^{\prime \prime }_{{\text{k}}}  \left( y \right) & = \frac{{\left( {12.6{ } + { }12.6} \right)}}{0.9} \\ & = 28 \\ \end{aligned} $$

$$ {\text{w}}^{\prime \prime }_{{\text{k}}}  \left( y \right) = \left( {{2}0.{89},{28}} \right)\quad {\text{So}}\;new\;{\text{w}}_{{\text{k}}} {\prime \prime } = \left( {{2}0.{89},{28}} \right). $$

**Figure 6:**
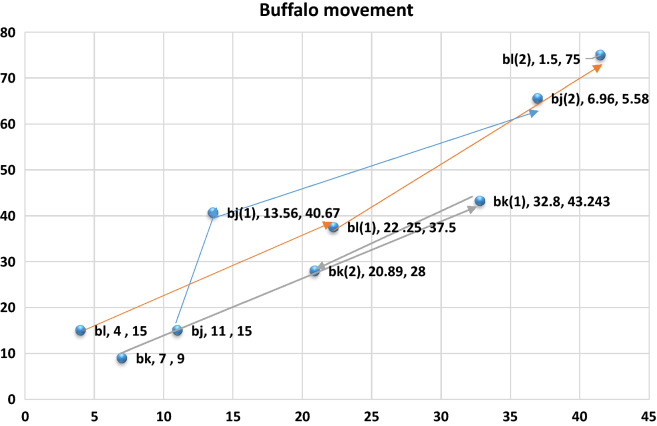
2nd Iteration.

Next, we plot this present location of our buffalo into our graph shows that the buffalo is migrating towards our global maximum. (see Fig. 6):

*Iteration 2 (for* $$b_{j}$$*):*

$bp$ = each buffalo’s previous points, $bg$ = 32.8,43.2 ($b_{k } (1)$), picked randomly

$$ {\text{m}}^{\prime \prime }_{{\text{j}}}  = {\text{m}}_{{\text{j}}} {\prime } + {\text{lp}}1\left( {bg{-}{\text{w}}_{{\text{j}}} } \right) + {\text{lp}}2\left( {bp.{\text{j}} - {\text{w}}_{{\text{j}}} } \right) $$

$$ \begin{aligned} {\text{m}}^{\prime \prime }_{{\text{j}}} \left( x \right) & = {8}.{2} + 0.6\left( {{32}.{8}{-}{13}.{56}} \right) + 0.5*\left( {{6}.{1}{-}{13}.{56}} \right) \\ & = {8}.{2} + 0.6\left( {{19}.{24}} \right) + 0.5\left( { - {7}.{46}} \right) \\ & = {8}.{2} + {11}.{54} + - {3}.{73} \\&{\text{m}}^{\prime \prime }_{{\text{j}}} \left( x \right) = {16}.0{1}\\ \end{aligned} $$

$$ {\text{m}}^{\prime \prime }_{{\text{j}}}  = {\text{m}}_{{\text{j}}} {\prime } + {\text{lp}}1\left( {bg{-}{\text{w}}_{{\text{j}}} } \right) + {\text{lp}}2\left( {bp.{\text{j}} - {\text{w}}_{{\text{j}}} } \right) $$

$$ \begin{aligned} {\text{m}}^{\prime \prime }_{{\text{j}}} \left( y \right) & = {21}.{6} + 0.6\left( {{43}.{2} - {4}0.{67}} \right) + 0.5\left( {{18}.{3} - {4}0.{67}} \right) \\ & = {21}.{6} + 0.6\left( {{2}.{53}} \right) + 0.5\left( { - {22}.{37}} \right) \\ & = {21}.{6} + {1}.{38} + - {11}.{19} \\ & = {11}.{79} \\ \end{aligned} $$

$$ {\text{m}}^ {\prime \prime }_{{\text{j}}}\left( y \right) = new\;{\text{m}}_{{\text{j}}} = \left( {{16}.0{1},{11}.{79}} \right). $$

Plotting the present ${\text{m}}_{{\text{j}}}$ values (16.01, 11.79) to Eq. (2):

$$ {\text{w}}^{\prime \prime }_{{\text{j}}} = \frac{{\left( {{\text{w}}_{{\text{j}}} + {\text{m}}_{{\text{j}}} } \right)}}{\lambda } $$

$$ \begin{aligned} {\text{w}}^{\prime \prime }\left( x \right) & = \frac{{\left( {13.56 + { }16.01} \right)}}{0.8} \\ & = {36}.{96} \\ \end{aligned} $$

$$ \begin{aligned} {\text{w}}^{\prime \prime }_{{\text{j}}}\left( y \right) & = \frac{{\left( {40.67{ } + { }11.79} \right)}}{0.8} \\ & = {65}.{58}\quad {\text{w}}_{{\text{j}}} = \left( {{36}.{96},{65}.{58}} \right) \\ \end{aligned} $$

Now $$ b_{j}$$ has moved to 36.96, 65.58 (Fig. 5).

*Iteration 2 (for* $$b_{l}$$*):*

$bp$ = each buffalo’s starting points, $bg$ = 32.8,43.2 ($b_{k } (1)$), picked randomly

$$ {\text{m}}^{\prime \prime }_{{\text{l}}} = {\text{m}}_{{\text{l}}} {\prime }{ } + {\text{lp}}1\left( {bg{-}{\text{w}}_{{\text{l}}} } \right) + {\text{lp}}2\left( {bp.{\text{l}} - {\text{w}}_{{\text{j}}} } \right) $$

$$ \begin{aligned} {\text{m}}^{\prime \prime }_{{\text{l}}}\left( x \right) & = {6}.{8} + - 0.6\left( {{32}.{8}{-}{22}.{25}} \right) + 0.5*\left( {{11}{-}{22}.{25}} \right) \\ & = {6}.{8} + 0.6\left( {{1}0.{55}} \right) + 0.5\left( { - {11}.{25}} \right) \\ & = {6}.{8} + {6}.{3} + - {5}.{63} \\ & {\text{m}}^{\prime \prime }_{{\text{l}}}\left( x \right) = {7}.{47} \\\end{aligned} $$

$$ {\text{m}}^{\prime \prime }_{{\text{l}}} = {\text{m}}_{{\text{l}}} {\prime } + {\text{lp}}1\left( {bg{-}{\text{w}}_{{\text{l}}} } \right) + {\text{lp}}2\left( {bp.{\text{l}} - {\text{w}}_{{\text{j}}} } \right) $$

$$ \begin{aligned} {\text{m}}^{\prime \prime }_{{\text{l}}}\left( y \right) & = {15} + 0.6\left( {{43}.{2} - {15}} \right) + 0.5\left( {{15} - {37}.{5}} \right) \\ & = {15} + 0.6\left( {{28}.{2}} \right) + 0.5\left( { - {22}.{5}} \right) \\ & = {15} + {16}.{92} + - {11}.{25} \\ \end{aligned} $$

$$ {\text{m}}^{\prime \prime }_{{\text{l}}} \left( y \right) = {2}0.{67}\quad new\;{\text{m}}_{l} = \left( {{7}.{47},{2}0.{67}} \right). $$

Applying the present values of ${\text{m}}_{l}$ (7.47, 20.67) to Eq. (2):

$$ {\text{w}}^{\prime \prime }_{{\text{l}}} = \frac{{\left( {{\text{w}}_{{\text{l}}} + {\text{m}}l} \right)}}{\lambda } $$

$$ \begin{aligned} {\text{w}}^{\prime \prime }_{{\text{l}}}\left( x \right) & = \frac{{\left( {22.25 + { }6.8} \right)}}{0.7} \\ & = 41.5 \\ \end{aligned} $$

$$ \begin{aligned} {\text{w}}^{\prime \prime }_{{\text{l}}}\left( y \right) & = \frac{{\left( {37.5{ } + { }15} \right)}}{0.7} \\ & = {75}\quad {\text{new}}\;{\text{w}}_{{\text{l}}} = \left( {{41}.{5},{ 75}} \right) \\ \end{aligned} $$

At this point, the ABO verifies the exit criterion and discovers that this has been met since its assignment is to obtain the optimum result which is (41.5, 75).

## Implementation of the ABO and the CS

In this section, the popular benchmark Rosenbrock function is implemented using ABO and Cuckoo Search on a PC, 4 GB RAM, Intel Duo Core i7 370 CPU @ 3.40 GHz, 3.40GH running Windows 10 using MATLAB 2012b.The ABO parameters used in the experiments are lp1 = 0.7; lp2 = 0.5. For the CS, the parameters are: step = u./abs (v). ^ (1/beta); step size = 0.01* step; u = rand (size (s)) * sigma; pa = 0.5; v = rand (size(s)). Each specific experimental was executed five times.

The aim of the experimental evaluations is to unravel the effect of the number of buffalos/nests and the iterations in obtaining good results with the objective of performing a comparative performance evaluation of both algorithms. The choice of Cuckoo Search is borne out of the fact that Cuckoo Search, in addition to being a recently developed metaheuristic, has so far proven to be very efficient and effective in solving several optimization problems. Some of the successful application areas of the Cuckoo Search includes job scheduling, flow shop scheduling, travelling salesman’s problems, image processing, speech recognition, global optimization problems etc^43^.

The evaluation metrics used in the comparative performance analysis in this study are the algorithms efficiency and effectiveness. Effectiveness as used in this study refers to the capacity of the algorithms to algorithms to obtain the optimal results while algorithm efficiency refers to the algorithms capacity to obtain results using the most optimized resources^44,45^.

### Cuckoo search

**Figure 7:**
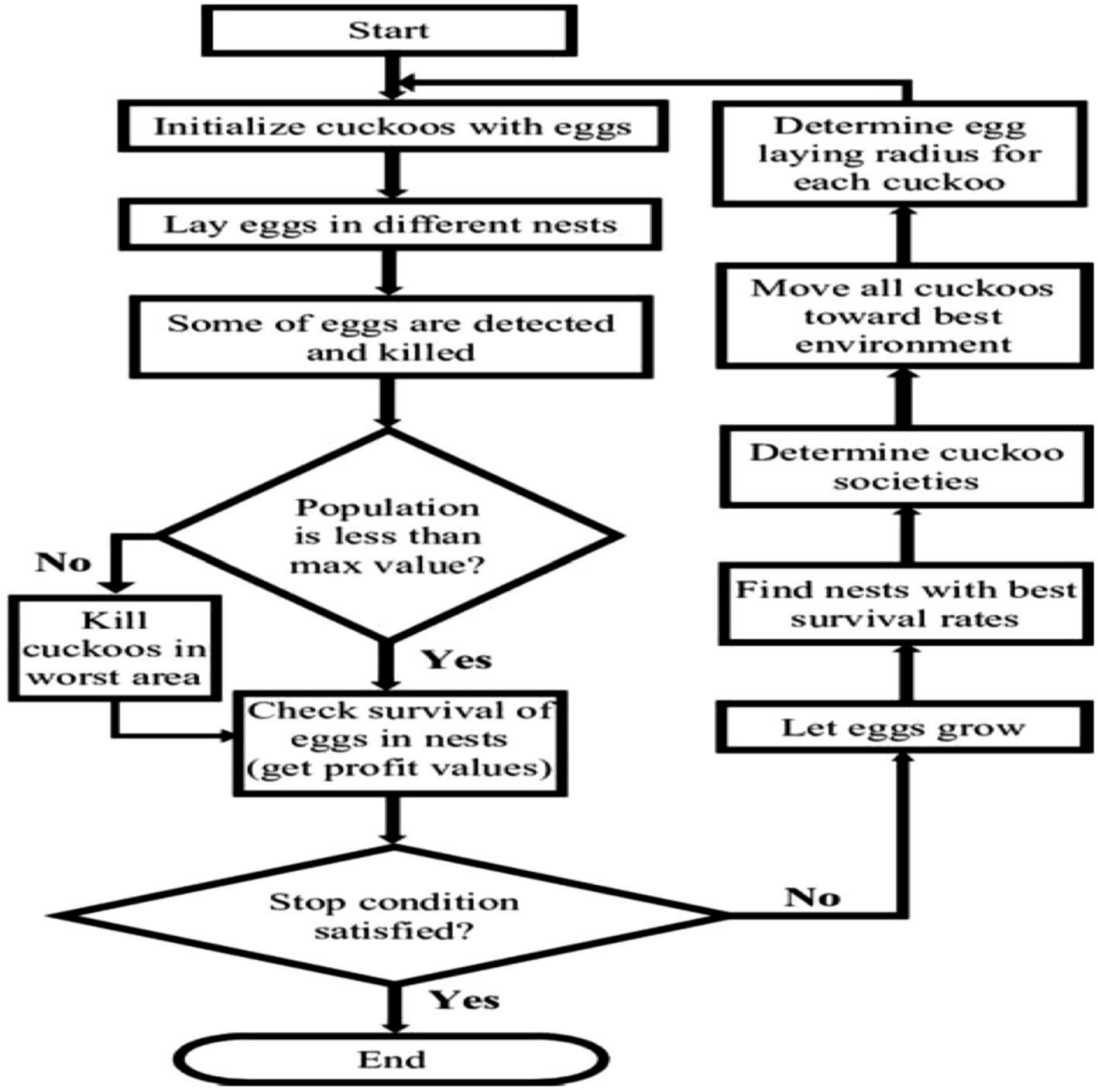
CS pseudocode.


Cuckoo Search (CS) algorithm was designed by X. Yang and S. Deb. The algorithm is a mathematical simulation of the irresponsible behaviour of cuckoo birds brooding over their eggs until such eggs are hatched^46^. The CS pseudocode^47^ and flowchart^48^ is presented in Fig. 7:

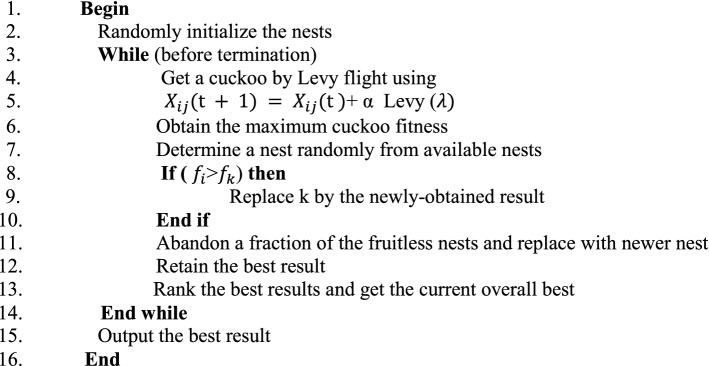


CS starts its search by initializing, randomly, cuckoo bird nests. Next using Eq. (3), it evaluates the fitness of each nest.

3 $$ X_{ij} \left( {{\text{t}} + 1} \right) = X_{ij} \left( {\text{t }} \right) + \upalpha {\text{Levy}}(\lambda ) $$

Once it obtains the cuckoo nest with the best fitness, it records same and compares it with the next best nest fitness in the subsequent iterations. In each iteration, the CS algorithm verifies the stopping criteria. Once the algorithm reaches the stopping criteria, the algorithm outputs the best fitness as the answer to the optimization problem being solved.

The dataset for the Global optimization problems are obtained from the benchmark continuous global optimization test problems^49^. Rosenbrock function is one of functions designed by Kenneth Dejong for his Ph.D. Thesis in 1975. The others functions include Dejong 5 (Shekel foxhole), Sphere, Quartic and Step functions. Over time it has become very popular among researchers for testing optimization algorithms. Note that the benchmark Rosenbrock function is^50^:

4 $$ f\left( x \right) = \mathop \sum \limits_{i = 1}^{d - 1} \left[ {\left( {100 x_{i} - x_{i}^{2} } \right)^{2} + \left( {x_{i} - 1} \right)^{2} } \right] $$

and the optimum solution is:

5 $$ f\left( x \right) = 0 $$

### Comparative performance evaluation of ABO and cuckoo search

The experimental outcome when 10 buffalos/nests are executed with varying buffalo and nests population deployed to the search space is presented in Table 1. The choice of 10 buffalos/nest was informed by the recommendation of the designer of Cuckoo Search that the algorithms works better when a population of between 15 and 40 nests are used^51^. The experimental outcome is presented in Table 1.

**Table 1:** Simulation output with 10 buffalos/nests with different iterations.

Iterations	ABO				CS			
	${f}_{min}$	Average	Time (s)	Average time (s)	${f}_{min}$	Average	Time (s)	Average Time (s)
10	0.0359	0.0426	0.022	0.021	2.3080	0.5634	0.040	0.031
	0.0580		0.021		0.0013		0.031	
	0.0028		0.022		0.0329		0.033	
	0.1025		0.021		0.4738		0.034	
	0.0137		0.019		0.0012		0.018	
100	0.0018	0.0527	0.030	0.0542	3.9731 ${e}^{-13}$	5.0611 ${e}^{-13}$	0.172	0.163
	0.0018		0.059		9.8137 ${e}^{-14}$		0.162	
	0.0031		0.060		4.4142 ${e}^{-12}$		0.154	
	0.0308		0.059		1.5596 ${e}^{-13}$		0.170	
	0.2259		0.063		5.5449 ${e}^{-15}$		0.157	
1000	0.0011	0.0077	0.468	0.4650	4.6147 ${e}^{-85}$	4.4527 ${e}^{-78}$	1.565	1.5874
	0.0004208		0.455		1.0024 ${e}^{-83}$		1.556	
	0.00065974		0.472		4.2801 ${e}^{-78}$		1.563	
	0.0124		0.464		4.2170 ${e}^{-69}$		1.600	
	0.0241		0.466		8.1493 ${e}^{-75}$		1.653	
5000	2.503 ${e}^{-07}$	2.9357 ${e}^{-06}$	2.293	2.273	0	2.4697 ${e}^{-320}$	8.118	7.9480
	1.0443 ${e}^{-04}$		2.291		3.0304 ${e}^{-318}$		8.086	
	1.4071 ${e}^{-06}$		2.277		4.3782 ${e}^{-318}$		7.867	
	6.6688 ${e}^{-06}$		2.247		0		7.848	
	3.0553 ${e}^{-05}$		2.258		4.9407 ${e}^{-324}$		7.821	
10,000	1.8499 ${e}^{-05}$	3.7547 ${e}^{-05}$	4.640	4.488	0	0	15.372	15.761
	3.5446 ${e}^{-05}$		4.497		0		15.586	
	2.8062 ${e}^{-05}$		4.421		0		15.683	
	6.9589 ${e}^{-05}$		4.422		0		15.979	
	3.6141 ${e}^{-05}$		4.458		0		16.183	

The simulation results in Table 1 indicate that the best average outcome of the ABO (2.9357 ${e}^{-06}$ at an average of 2.273 s) was obtained at 5000 iterations when searching with 10 buffalos. It is interesting, however, to note that a single best performance 2.503 ${e}^{-07}$ which is the best individual result was obtained when 10 buffalos are deployed using different iterations (10, 100, 1000, 5000 and 10,000). This is a mark of the algorithm’s randomness: a mark of good algorithms^50^.

In this Table, it is remarkable that the average output derived from using 10,000 iterations (3.7547 ${e}^{-05}$) is inferior to deploying just 5000 iterations (2.9357 ${e}^{-06}$). This result is interesting because in an earlier study on the Genetic Algorithm, it was observed that the use of a larger population/iteration leads to better results^52^. In the light of the findings of this study, it may be safe to say that the assertion of the earlier study could be algorithm-specific or problem-specific.

In terms of the comparative results, the ABO was discovered to be a better starter. For instance at 10 iterations, the ABO has a better result of 0.0426 compared to CS’s 0.5634. Similarly, the ABO was faster at this point with 0.021 than the CS’s 0.031.

However, in terms of obtaining the optimal or near optimal results, the tide changed from the 100 iterations, On the average, the CS, had better good results right from 100 iterations all through the 10,000 iterations but at a very inferior time when compared to the results obtained by the ABO. Right from when both algorithms were executed with just 100 iterations, the results of the CS were superior to those of the ABO. The average result of the CS at 100 iterations was 5.0611 ${e}^{-13}$ as opposed to ABO’s 0.0527. This trend continues to 10,000 when the CS obtained the optimal solution of 0.

Based on the findings of Table 1, it is necessary to investigate the performance of ABO and CS when a population of 50 search agents are deployed. This is the focus of the second set of experiments whose outcome is presented in Table 2.

**Table 2:** Simulation output with 50 buffalos/nests with different iterations.

Iterations	ABO				CS			
	${f}_{min}$	Average	Time (s)	Average time (s)	${f}_{min}$	Average	Time (s)	Average time (s)
10	0.0101	0.0357	0.063	0.051	0.1048	1.0334	0.071	0.0784
	0.0518		0.052		1.1899		0.068	
	0.091		0.051		0.0314		0.068	
	0.013		0.037		2.9252		0.068	
	0.0127		0.052		0.9157		0.117	
100	0.0126	0.0058	0.229	0.228	4.1464 ${e}^{-14}$	3.8376 ${e}^{-15}$	0.172	0.163
	0.0013		0.225		6.8572 ${e}^{-14}$		0.162	
	0.0044		0.229		2.6223 ${e}^{-16}$		0.154	
	0.0036		0.230		2.7811 ${e}^{-15}$		0.170	
	0.0069		0.227		2.7811 ${e}^{-16}$		0.157	
1000	6.1493 ${e}^{-05}$	4.4423 ${e}^{-04}$	1.981	2.032	1.3143 ${e}^{-54}$	2.2048 ${e}^{-55}$	6.231	6.310
	6.1548 ${e}^{-05}$		1.986		5.4190 ${e}^{-56}$		6.257	
	3.4435 ${e}^{-04}$		2.056		1.6071 ${e}^{-56}$		6.364	
	2.8068 ${e}^{-04}$		2.052		1.0727 ${e}^{-55}$		6.384	
	3.6573 ${e}^{-05}$		2.083		1.6111 ${e}^{-54}$		6.313	
5000	4.1663 ${e}^{-06}$	2.7808 ${e}^{-06}$	4.526	8.761	6.8572 ${e}^{-161}$	4.9646 ${e}^{-165}$	26.775	30.042
	1.9063 ${e}^{-07}$		9.830		2.6223 ${e}^{-169}$		25.366	
	2.7224 ${e}^{-05}$		9.737		2.7811 ${e}^{-165}$		31.439	
	1.6645 ${e}^{-05}$		10.012		7.3016 ${e}^{-165}$		32.744	
	3.4444 ${e}^{-06}$		9.702		5.2609 ${e}^{-167}$		33.884	
10,000	7.8032 ${e}^{-06}$	4.1005 ${e}^{-06}$	20.117	20.237	2.0759 ${e}^{-269}$	5.3958 ${e}^{-272}$	67.500	68.441
	5.9999 ${e}^{-07}$		20.734		6.5280 ${e}^{-272}$		68.044	
	2.0300 ${e}^{-07}$		19.853		8.5684 ${e}^{-271}$		68.585	
	1.0420 ${e}^{-06}$		20.138		8.2255 ${e}^{-269}$		70.631	
	3.6275 ${e}^{-05}$		20.341		1.5813 ${e}^{-279}$		67.447	

The simulation outcome of Table 2 follows the trend of Table 1. CS, generally, had better results than ABO but ABO has again proven to be a faster algorithm. A major contribution of this study is the discovery that the CS obtained better output at 5000 iterations when 10 nests were deployed (2.4697 ${e}^{-320}$) than when 50 nests (4.9646 ${e}^{-165}$). At 10,000 iterations using 10 nests, the CS converged at the optimum result (average: 0) but was unable to do same when 50 nests were used at the same 10,000 iterations (average: 5.3958 ${e}^{-272}$). Also one wonders why the need of as much as 10,000 iterations using 50 nests when the result of 10 nests with 5000 iterations could do a better job.

A closer look at the CS performance at just 1000 iterations shows that the algorithm performed better using just 10 nests at an average of 4.4527 ${e}^{-78}$ than when searching with 50 nests (average: 2.2048 ${e}^{-55}$). The finding here is, again, a deviation from the view that the more the iterations cum population, the better the result. When searching with 50 buffalos, the ABO had a better result at 5000 iterations with an average outcome of 2.7808 ${e}^{-06}$ than at 10,000 iterations with an average of 4.1005 ${e}^{-06}$. Aside these few exceptions, the other results follow the general trend that the more the population cum iterations, the more likely, the chance of a better result^53^.

In terms of the speed of execution, the experimental outcome in Table 2 is consistent with earlier findings that the deployment of more populations and more iterations may likely lead to more processing time but with better results^54^. The slow speed whenever more iterations and more populations are deployed is because the algorithm’s convergence gets slower as a result of more evaluations arising from such a situation.

## Comparative performance of ABO and flower pollination algorithm

Flower Pollination Algorithm (FPA) which was designed by X.S. Yang has been very exceptional in obtaining good results when applied to solve optimization problems. The algorithm inspired by the normal pollination of natural flowers through self-pollination (biotic pollination) by water or wind or cross pollination (abiotic pollination) by other animals uses levy flight to arrive at solutions. In FPA self-pollination represents global search and cross pollination, local search. In either the local or global search, there exists a strict regulation by a switch search mechanism with a probability p ∈ [0, 1]. Global pollination is modelled by the following process:

6 $$ {\text{x}}_{{\text{i}}}^{{{\text{t}} + {1}}} = {\text{x}}_{{\text{i}}}^{{\text{t}}} + {\text{L}}\left( {{\text{x}}_{{\text{i}}}^{{\text{t}}} {-}{\text{g}}_{{{\text{best}}}} } \right) $$

x_i_^t^ is the pollen i or solution vector x_i_ at iteration *t*. g_best_ is the globally best solution. The parameter L is a step size, and is drawn from a Lévy distribution.
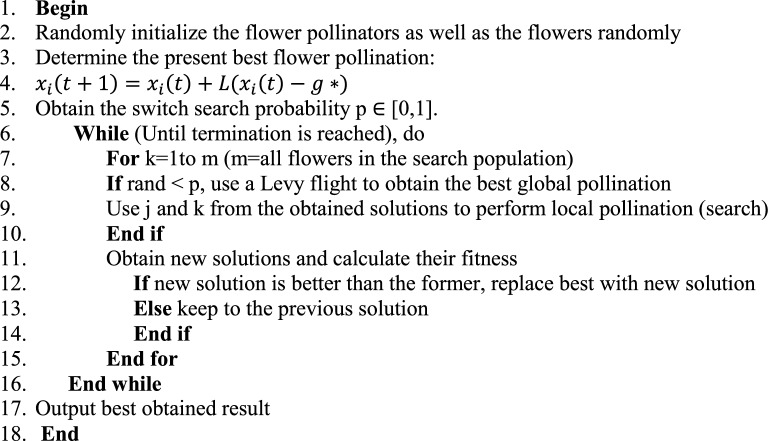


Table 3 below presents the comparative performance evaluation of the ABO and FPA.

**Table 3:** Simulation output of ABO and FPA using a population of 10 buffalos/flowers.

Iteration	Population	ABO		FPA	
		${f}_{min}$	Time (s)	${f}_{min}$	Time (s)
**10**	10	13.9122	0.243	40.3190	0.025
		22.3328	0.092	60.1138	0.025
		3.3325	0.075	19.2325	0.026
		11.8802	0.075	11.2907	0.027
		12.6747	0.074	18.9458	0.029
**Average**		**12.827**	**0.112**	**29.980**	**0.026**
**20**		12.6705	0.128	12.6705	0.068
		12.6705	0.128	12.6708	0.039
		12.6705	0.129	12.6705	0.039
		12.6705	0.127	12.6705	0.036
		12.6705	0.129	12.6705	0.037
**Average**		**12.6705**	**0.128**	**12.6706**	**0.044**
**100**		12.6705	0.574	2.5526	0.173
		7.8740	0.568	1.0754	0.124
		5.7373	0.572	4.3196	0.136
		6.6766	0.581	1.0014	0.125
		1.1096	0.569	1.0034	0.125
**Average**		**6.814**	**0.573**	**1.991**	**0.137**
**1000**		2.0259	5.528	3.4697	1.146
		3.8699	5.539	0.9961	1.126
		0.9980	5.521	0.9982	1.143
		10.8268	5.488	3.2507	1.134
		9.6829	5.524	1.0724	1.134
**Average**		**5.481**	**5.520**	**1.957**	**1.137**
**5000**		4.9505	27.466	0.9986	5.550
		2.0291	27.639	0.9983	5.565
		0.9980	27.347	0.9981	5.538
		2.9821	27.395	1.0036	5.575
		0.9992	27.395	1.0042	5.517
**Average**		**2.392**	**27.448**	**1.001**	**5.549**
**10,000**		0.9980	54.655	0.9992	11.097
		0.9980	54.861	0.9983	11.035
		0.9983	55.299	0.9980	11.316
		0.9980	55.129	0.9980	11.335
		0.9981	54.914	0.9984	11.096
**Average**		**0.9981**	**54.972**	**0.9984**	**11.176**

Significant values are in bold.

FPA pseudo code of FPA is presented:^10^.

In solving the complex Shekel Foxhole function that has 25 local minima and a global minimum, many algorithms struggle, but not FPA and ABO. Clearly, these two algorithms have been very competitive. Both algorithms used a population of 10 buffalos/flowers in their search which usually produces good results^2,3,29,47^. This population produced very good results as can be seen in Table 3. Please note that global minimum of the Shekel Foxhole function is^55^:

7 $$ x* = 0.9980 $$

From Table 3, it can be seen that the ABO arrived at the global optimum at iteration 1000. In the same vein, FPA best result at iteration1000 was 0.9982 which is also a good result. ABO’s average after five runs at iteration 5000 was 0.9981 to FPA’s 0.9984. It is obvious that the ABO has a slight advantage over the FPA in algorithm effectiveness. In terms of efficiency, FPA clearly performed better right from the beginning to the end. For instance while it took the ABO an average of 54.972 s to make five runs at 5000, the FPA only took 11.176. For emphasis, note that algorithm effectiveness is the capacity of algorithms to arrive at the global optimum but efficiency refers to the algorithm’s capacity to minimize the use of computer resources. Since the amount of time spent to arrive at a solution correlates with use of computer resources, the amount of time taken to arrive at a solution dictates the length of time that the computer resources are engaged: the shorter the time, the better^44^.

In all, from the foregoing analysis, since algorithm’s performance is judged primarily by its efficiency and effectiveness, in view of the competitive results posted by the ABO so far, the algorithm can be deemed a worthy inclusion to the body of swarm optimization algorithms in literature.

## Conclusion

This paper explains the algorithmic flow and data generation procedure of the African Buffalo Optimization algorithm. First, using very simple language as much as possible cum basic mathematical description with detailed examples, this paper explains the workings of the ABO in a manual setting. In the second part (see “Implementation of the ABO and the CS” and “Comparative performance of ABO and flower pollination algorithm”), a MATLAB implementation of the ABO CS and the FPA were done to solve the benchmark Rosenbrock and Shekel Foxhole functions with particular emphasis on the effect of the search population and the number of iterations in obtaining good results. After a number of experimental evaluations, the study agrees, that to a large extent, the more the number of iterations cum population, the more likely the chance of a better outcome.

However, it must be observed that the more the population cum number of iterations, the more execution time taken to arrive at a solution. This is because the more the population cum iterations, the more the evaluations, therefore, leading to slower convergence^54^. It is our sincere belief that with this detailed explanation cum data description of the ABO, the research community will explore the search capacity of this novel algorithm in solving different types of optimization problems in science and engineering applications bearing in mind that so far swarm optimization algorithms have been applied to so many real life problems. Some of the potential real life application areas of the African Buffalo Optimization algorithm include such as parameter estimations^56^, nonlinear system identification^57^ learning of the weights of neural networks^58^ and many others..

### Threats to validity

While we celebrate the good and competitive results of the algorithms used in this study, it is worthy of note, however, that good results could be a function of the machine used in the study, the programming language used for coding and implementation as well as technical expertise of the programmer. Another factor that could be a threat to the validity of results could be the choice of benchmark test cases. Those algorithms perform very well in the chosen benchmark cases may not be a guarantee that they will do the same in other benchmarks. Moreover, the choice of the algorithms used in this particular study could be a threat: while acknowledging that the algorithms used in this study performed well against one another, they may not do the same when compared with other algorithms. Nevertheless, that these threats to the validity of the claims made in this study are highlighted, does not, in any way, contradict the findings/results obtained. For further study, it is recommended that performance of these algorithms be applied to a different dataset such as the CEC recent functions as well as similar datasets.

## References

[1] Odili JB (2018). The dawn of metaheuristic algorithms. Int. J. Softw. Eng. Comput. Syst..

[2] Odili J, Kahar MNM, Noraziah A, Kamarulzaman SF (2017). A comparative evaluation of swarm intelligence techniques for solving combinatorial optimization problems. Int. J. Adv. Rob. Syst..

[3] Odili, J. B., Kahar, M. N. M., Noraziah, A., Zarina, M. & Haq, R. U. Performance analyses of nature-inspired algorithms on the traveling salesman’s problems for strategic management. *Intell. Autom. Soft Comput.* 1–11 (2017).

[4] Yıldız A, Pholdee N, Bureerat S, Yıldız AR, Sait SM (2020). Sine-cosine optimization algorithm for the conceptual design of automobile components. Mater. Test..

[5] Odili JB (2017). Combinatorial optimization in science and engineering. Curr. Sci..

[6] Odili JB, Noraziah A, Ambar R, Abd Wahab MH (2018). Implementation strategies for the cuckoo search and the African buffalo optimization for the benchmark Rosenbrock function. Eurasia Proc. Sci. Technol. Eng. Math..

[7] Panagant N, Pholdee N, Bureerat S, Yildiz AR, Mirjalili S (2021). A comparative study of recent multi-objective metaheuristics for solving constrained truss optimisation problems. Arch. Comput. Methods Eng..

[8] Noraziah, J. B. O. A. & Abd Wahab, M. H. African Buffalo optimization algorithm for collision-avoidance in electric fish.

[9] Odili, J. B. & Kahar, M. M. in *National Conference for Postgraduate Research, Universiti Malaysia Pahang*, Vol. 641–648.

[10] Yang, X.-S. in *International Conference on Unconventional Computing and Natural Computation.* 240–249 (Springer).

[11] Khari M, Kumar P, Burgos D, Crespo RG (2018). Optimized test suites for automated testing using different optimization techniques. Soft. Comput..

[12] Vimal S (2020). Energy enhancement using Multiobjective Ant colony optimization with Double Q learning algorithm for IoT based cognitive radio networks. Comput. Commun..

[13] Odili JB, Noraziah A (2018). African buffalo optimization for global optimization. Curr. Sci.

[14] Oyelade, O. N. & Ezugwu, A. E. Ebola optimization search algorithm (EOSA): A new metaheuristic algorithm based on the propagation model of Ebola virus disease. arXiv:2106.01416 (2021).

[15] Abualigah L, Abd Elaziz M, Sumari P, Geem ZW, Gandomi AH (2022). Reptile search algorithm (RSA): A nature-inspired meta-heuristic optimizer. Expert Syst. Appl..

[16] Yıldız BS (2017). Natural frequency optimization of vehicle components using the interior search algorithm. Mater. Test..

[17] Agushaka JO, Ezugwu AE, Abualigah L (2022). Dwarf mongoose optimization algorithm. Comput. Methods Appl. Mech. Eng..

[18] Chiclana F (2018). ARM–AMO: An efficient association rule mining algorithm based on animal migration optimization. Knowl.-Based Syst..

[19] Abed-alguni BH, Alawad NA (2021). Distributed Grey Wolf Optimizer for scheduling of workflow applications in cloud environments. Appl. Soft Comput..

[20] Karagöz S, Yıldız AR (2017). A comparison of recent metaheuristic algorithms for crashworthiness optimisation of vehicle thin-walled tubes considering sheet metal forming effects. Int. J. Veh. Des..

[21] Yıldız BS, Yıldız AR (2019). The Harris hawks optimization algorithm, salp swarm algorithm, grasshopper optimization algorithm and dragonfly algorithm for structural design optimization of vehicle components. Mater. Test..

[22] Yıldız BS, Yıldız AR (2018). Comparison of grey wolf, whale, water cycle, ant lion and sine-cosine algorithms for the optimization of a vehicle engine connecting rod. Mater. Test..

[23] Mohamed AW, Hadi AA, Mohamed AK (2019). Gaining-sharing knowledge based algorithm for solving optimization problems: A novel nature-inspired algorithm. Int. J. Mach. Learn. Cybern..

[24] Odili JB, Kahar MNM, Noraziah A (2019). African buffalo optimization algorithm for tuning parameters of a PID controller in automatic voltage regulators. Int. J. Simul: Syst. Sci. Technol..

[25] Odili JB, Kahar MNM, Noraziah A (2017). Parameters-tuning of PID controller for automatic voltage regulators using the African buffalo optimization. PLoS ONE.

[26] Odili, J. B., Noraziah, A. & Sidek, R. M. in *IOP Conference Series: Materials Science and Engineering.* 012030 (IOP Publishing).

[27] Hassan, M. H. *et al.* Integrating African Buffalo optimization algorithm in AODV routing protocol for improving the QoS of MANET. *J. Southwest Jiaotong Univ. ***54** (2019).

[28] Singh P, Meena NK, Slowik A, Bishnoi SK (2020). Modified african buffalo optimization for strategic integration of battery energy storage in distribution networks. IEEE Access.

[29] Odili JB, Noraziah A, Babalola AE (2020). Flower Pollination Algorithm for data generation and analytics-a diagnostic analysis. Sci. Afr..

[30] Odili JB, Noraziah A, Babalola AE (2022). A new fitness function for tuning parameters of Peripheral Integral Derivative Controllers. ICT Express.

[31] Odili JB, Noraziah A, AbdWahab MH (2020). African buffalo optimization algorithm for collision-avoidance in electric fish. Intell. Autom. Soft Comput..

[32] Logg JM, Minson JA, Moore DA (2019). Algorithm appreciation: People prefer algorithmic to human judgment. Organ. Behav. Hum. Decis. Process..

[33] Lorenzen E, Heller R, Siegismund HR (2012). Comparative phylogeography of African savannah ungulates. Mol. Ecol..

[34] Jori F (2011). A questionnaire-based evaluation of the veterinary cordon fence separating wildlife and livestock along the boundary of the Kruger National Park, South Africa. Prevent. Vet. Med..

[35] Odili JB, Kahar MNM (2015). African buffalo optimization (ABO): A new meta-heuristic algorithm. J. Adv. Appl. Sci..

[36] Odili JB, Kahar MN, Noraziah A (2016). Solving traveling salesman’s problem using African buffalo optimization, honey bee mating optimization & Lin-Kerninghan algorithms. World Appl. Sci. J..

[37] Jones CB (1983). Tentative steps toward a development method for interfering programs. ACM Trans. Program. Lang. Syst. (TOPLAS).

[38] Jakeman AJ, Letcher RA, Ten Norton JP (2006). iterative steps in development and evaluation of environmental models. Environ. Model. Softw..

[39] Baritompa B, Hendrix EM (2005). On the investigation of stochastic global optimization algorithms. J. Global Optim..

[40] Odili, J. B. & Fatokun, J. O. in *2020 International Conference in Mathematics, Computer Engineering and Computer Science (ICMCECS).* 1–8 (IEEE).

[41] Odili JB, Mohmad Kahar MN (2016). African buffalo optimization. Int. J. Softw. Eng. Comput. Syst..

[42] Aydoğdu İ, Akın A, Saka M (2016). Design optimization of real world steel space frames using artificial bee colony algorithm with Levy flight distribution. Adv. Eng. Softw..

[43] Kamat, S. & Karegowda, A. A brief survey on cuckoo search applications. *Int. J. Innovative Res. Comput. Commun. Eng.* **2** (2014).

[44] Odili JB, Noraziah A, Zarina M (2021). A comparative performance analysis of computational intelligence techniques to solve the asymmetric travelling salesman problem. Comput. Intell. Neurosci..

[45] Odili JB, Noraziah A, Ambar R, AbdWahab MH, Fakheraldin M (2018). Teaching computer science in the universities in third world countries: Challenges. Eurasia Proc. Educ. Soc. Sci..

[46] Yang, X.-S. & Deb, S. in *World Congress on Nature & Biologically Inspired Computing, 2009. NaBIC 2009.* 210–214 (IEEE).

[47] Odili JB (2017). Implementation analysis of cuckoo search for the benchmark rosenbrock and levy test functions. J. Inf. Commun. Technol..

[48] Majidi, M., Ozdemir, A. & Ceylan, O. in *2017 19th International Conference on Intelligent System Application to Power Systems (ISAP).* 1–6 (Ieee).

[49] Ali MM, Khompatraporn C, Zabinsky ZB (2005). A numerical evaluation of several stochastic algorithms on selected continuous global optimization test problems. J. Global Optim..

[50] Khompatraporn C, Pintér JD, Zabinsky ZB (2005). Comparative assessment of algorithms and software for global optimization. J. Global Optim..

[51] Mareli M, Twala B (2018). An adaptive Cuckoo search algorithm for optimisation. Appl. Comput. Inform..

[52] Tsoy, Y. R. in* The 7th Korea-Russia International Symposium on Science and Technology, 2003. Proceedings KORUS 2003.* 181–187 (IEEE).

[53] Raferty A, Lewis S (1995). The number of iterations, convergence diagnostics and generic Metropolis algorithms. Pract. Markov Chain Monte Carlo.

[54] Carr, J. An introduction to genetic algorithms. *Senior Project*, 1–40 (2014).

[55] Function, S. F. Shekel Foxhole Function. *Electric Power Systems Analysis and Nature-Inspired Optimization Algorithms*. https://al-roomi.org/benchmarks/unconstrained/2-dimensions/7-shekel-s-foxholes-function.

[56] Ammara M, Aneela Z, Ho LS, ur Rehman A, Zahoor RMA (2020). Integrated computational intelligent paradigm for nonlinear electric circuit models using neural networks, genetic algorithms and sequential quadratic programming. Neural Comput. Appl..

[57] Tariq HB (2021). Maximum-likelihood-based adaptive and intelligent computing for nonlinear system identification. Mathematics.

[58] Sabir, Z., Ali, M. R. & Sadat, R. Gudermannian neural networks using the optimization procedures of genetic algorithm and active set approach for the three-species food chain nonlinear model. *J. Ambient Intell. Hum. Comput.* 1–10 (2022). 10.1007/s12652-021-03638-3 PMC8763432 35069921

